# A randomized comparison of fluoroscopic techniques for implanting pacemaker lead on the right ventricular outflow tract septum

**DOI:** 10.1007/s10554-016-0840-1

**Published:** 2016-01-21

**Authors:** Dongli Chen, Huiqiang Wei, Jiaojiao Tang, Lie Liu, Shulin Wu, Chunying Lin, Qianhuan Zhang, Yuanhong Liang, Silin Chen

**Affiliations:** Department of Cardiology, Guangdong Cardiovascular Institute, Guangdong General Hospital, Guangdong Academy of Medical Sciences, Guangzhou, 510000 Guangdong China

**Keywords:** Alternative site pacing, Right ventricular outflow tract septal pacing, Fluoroscopy, Echocardiography

## Abstract

Right ventricular outflow tract (RVOT) septal pacing is commonly performed under the standard fluoroscopic positions during procedure. The aim of the prospective, randomized study was to evaluate the accuracy of the combination of standard fluoroscopic and left lateral (LL) fluoroscopic views for determination of RVOT septal position compared with standard fluoroscopic views alone. We prospectively enrolled patients who had indications for implantation of a permanent pacemaker. Patients were randomly assigned into two groups based on intraoperative fluoroscopic views as follows: LL group (three standard fluoroscopic views + LL fluoroscopic view) or standard group (three standard fluoroscopic views). Transthoracic echocardiography (TTE) determination of pacing sites was applied in all patients 3 days after pacemaker implantation. The implantation success rate of RVOT septal pacing was compared between groups. A total of 143 patients (59 males, mean age 57.6 ± 16.3 years) with symptomatic bradyarrhythmia were studied, of whom, 72 patients were randomized to LL group and 71 to standard group. TTE determination of pacing sites was compared with two groups. In the LL group, 60 patients (83 %) were achieved in RVOT septal position. In the standard group, however, the position of RVOT septum was only observed in 48 patients (68 %). The success rate of RVOT septal position in LL group was significantly higher than standard group (*p* = 0.029). Comparing to traditional views, combining LL view in the procedure will approve the accuracy of RVOT septal pacing site.

## Introduction

Pacing from the right ventricular (RV) apex induces abnormal electrical and mechanical activation patterns, which lead to detrimental effects on cardiac structure and pump function [1–3]. As a result, there is growing interest in alternative RV pacing sites. Among the different ventricular pacing sites, the most studied of alternative pacing sites has been the right ventricular outflow tract (RVOT) septum due to a more physiological pattern of ventricular activation [4, 5]. According to the current radiological criteria, documentation of RVOT septal position was acquired using three standard fluoroscopic views: posteroanterior (PA), 40° right anterior oblique (RAO), 40° left anterior oblique (LAO) [6]. The most important is the position of 40° LAO fluoroscopic view: RV lead is believed to be inserted into RVOT septal position if the lead faces toward the spine [7]. However, the efficacy and benefit of RVOT pacing are still controversial [8–10].

**Fig. 1:**
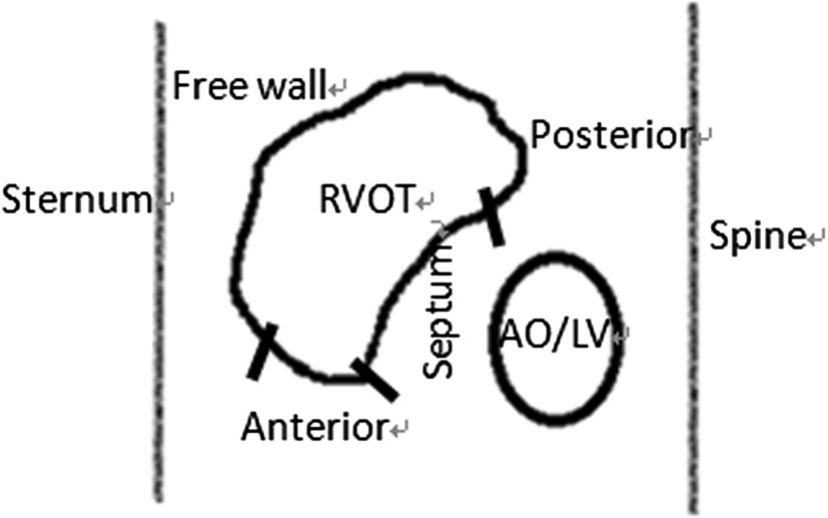
Cross-section of the chest from *left* leteral projection. The four areas of *right* ventricular outflow tract are schematically demonstrated

According to the anatomy described by Mond et al. [11], the RVOT is composed of four segments: septal, anterior, posterior and free walls. The septum lies posteriorly, the free wall in front, and between them is the anterior wall. Therefore, the 40° LAO fluoroscopic view is difficult to differentiate between RVOT septal and anterior wall positions. Several reports have suggested that the implantation success rate of true RVOT septum is far from satisfaction based on published radiological criteria [12–14]. The conventional fluoroscopic views seem to be sub-optimal and targeting the true RVOT septal pacing might be technically challenging. Left lateral (LL) fluoroscopic view appears to be valuable to indicate septal placement as it clearly defined the antero-posterior plane (Fig. 1) [11, 15]. But the view that may assist in confirming RVOT septum has not been proven. Accordingly, the aim of the present Aprospective, single-center, randomized study was to investigate the value of LL fluoroscopic view that confirm the RVOT septal position and differentiate this site from anterior and free walls, using two-dimensional transthoracic echocardiography (TTE) to validate pacing sites.

## Methods

### Patient population

The present study was a single-center randomized study performed from January 2013 to December 2014. Patients aged 18 years and older who had a standard indication for permanent pacemaker owing to symptomatic sick sinus syndrome or high degree atrioventricular block were included in this study. Patients were excluded before randomization if they met any of the following criteria: leads inserted into RV apex; indications for an implantable cardioverter defibrillator (ICD) or cardiac resynchronization therapy (CRT); clinical manifestations of congestive heart failure; chronic atrial fibrillation; moderate or greater degree of valvulopathy; chronic obstructive pulmonary disease; absence of informed content. All patients gave written consent to participate in the study before randomization. Patients were randomly assigned in a 1:1 ratio to two groups according to intraoperative fluoroscopic views: LL group (three standard fluoroscopic views + LL fluoroscopic view) or standard group (three standard fluoroscopic views). The randomization process was performed on the basis of numbered containers. The interventions (combined or not with use of LL fluoroscopic view) were sealed in the sequentially numbered opaque identical envelopes. The study protocol (20150814) was approved by the Institutional Ethics Committee.

### Pacemaker implantation procedure

**Fig. 2:**
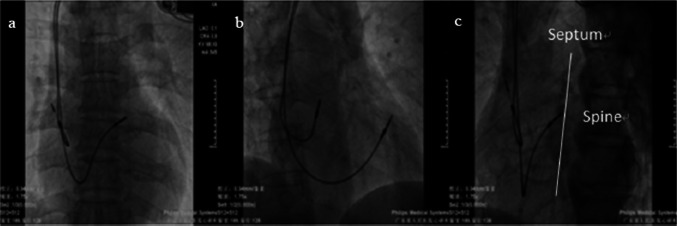
The conventional fluoroscopic images for lead implantation; **a** PA view: the pacemaker lead is in the RVOT position; **b** Thirty-degree RAO view; **c** Forty-degree LAO view: lead facing to the spine is septum

**Fig. 3:**
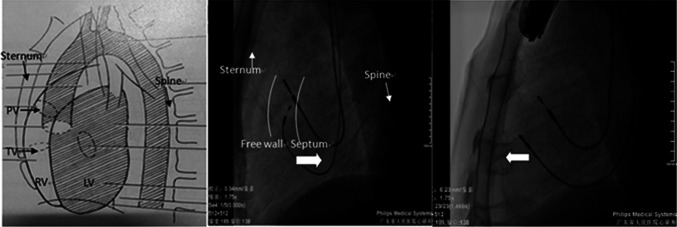
LL view of the heart (*left*); LL fluoroscopic image showing the lead tip in the RVOT septum (*mid*) projecting posteriorly and in the RVOT non-septum (*right*) projecting anteriorly

Single-chamber and double-chamber pacemaker systems were performed by a group of operators experienced in RVOT septal lead placement. Prophylactic intravenous antibiotics were given half an hour before the procedure. Pacemaker implantation procedure was done under local anesthesia. The right ventricular (RV) lead was inserted via the left- or right-side subclavian venous approach. Commercially available 58 cm 7—French bipolar steroid-eluting active fixation lead (Capsure-Fix Novus 5076, Medtronic Inc., Minneapolis, MN, USA or Tendril ST 1888TC, St.Jude Medical Inc., St.Paul, MN, USA) was used for RV septal implants. Leads were inserted into RVOT septal position using a standard hand-shaped stylet as previously described by Mond et al. [11]. The style was fashioned with generous curve and a terminal straight bend with posterior angulation. First, the lead was initially advanced into the pulmonary artery guided by the posterior-anterior (PA) position. Afterwards, it was withdrawn slowly until the tip of lead was placed below the pulmonary valve on the RVOT. The 40º right anterior oblique (RAO) projection was used to prevent inadvertent positioning in the coronary sinus and great cardiac vein. RVOT septal lead positioning was determined once the 40º left anterior oblique (LAO) fluoroscopic view showed the lead tip pointing to the spine in the standard group (Fig. 2). The position of the lead in the RVOT septum was also confirmed by fluoroscopy using the LL fluoroscopic position in the LL group before helix deployment. Orientation of the lead tip was classified as anterior or posterior in the LL projection. A posterior projection of the lead towards the spine indicated septal placement (Fig. 3). If the RV lead from the LL group met the criteria for RVOT septum in 40° LAO projection, but not in the LL fluoroscopic view, the lead was retracted and advanced again to the pulmonary artery and the maneuver repeated. If this was not successful, the stylet sometimes had to be reshaped in case of difficult lead positioning, as the curves tended to straighten with time.

Once the tip of the RV lead made attachment with RVOT setptal positioning, the screw was deployed. The ventricular stimulation threshold at a pulse width of 0.48 ms, R-wave amplitude and lead impedance measurements were taken several minutes after screw deployment. Perioperative complications requiring intervention were recorded.

### Determination of RVOT pacing sites by TTE

**Fig. 4:**
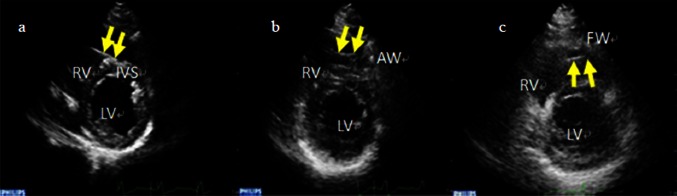
TTE determination of pacemaker lead position in parasternal short-axis view. The exact positions of the leads are documented (*yellow arrow*). **a** The lead is inserted into septum. **b** The lead passes over the septum and attaches into the anterior wall. **c** The position of the lead is anchored into the free wall

TTE presents an exact tool for assessing the exact anatomic location of pacing sites [12, 16, 17]. TTE was performed in all cases 3 days after pacemaker implantation by two observers who were blinded to the lead position. Disagreements between observers were resolved by consensus. Echocardiography was carried out with the subjects at rest in the left lateral decubitus position with a commercially available ultrasound transducer and equipment (S5-1 probe, Philips IE33, Ultrasound, Bothell, Washington, USA). 2D images were acquired from during end-expiratory held respiration and digitally stored at frame rates of 40–65 frames/second. The exact lead position, defined as the myocardium attachment site of the tip of RV lead, was documented using parasternal short-axis (PSAX) views. The correct location of the lead tip was the primary end point of the study. First, RVOT was displayed in PSAX views at the level of aortic valve. One part of the lead was seen within the RVOT. Then, the tip of RV lead was actively tracked using all available PSAX views. At last, the position of the lead was completely exposed and verified. We categorized the position of the leads into RVOT septum if the direction of the tip of the lead and its attachment site were seen to the plane of interventricular septum (Fig. 4).

### Electrocardiography analysis

A standard 12-lead ECG was recorded during forced ventricular pacing (VVI, 10 bpm above baseline ventricular rate) at a paper speed of 25 mm/s with chest and limb leads placed in standard positions. The ECG parameters derived from RVOT septal pacing and non-septal pacing were analyzed: (1) QRS duration, (2) presence of q-wave or negative QRS complex in lead I, (3) presence of QRS notching in the inferior leads, (4) QRS transition zone in the precordial leads. Transition zone was defined as the lead with R > (Q + S) amplitude.

### Statistical analysis

Continuous variables are expressed as mean value ± standard deviation. Categorical variables were compared by Chi square test. Continuous variables showing normal distribution according to the Shapiro–Wilk test and histogram analysis were compared using Student’s *t* test between two groups. The Mann–Whitney *U* test was performed for comparing groups if variables did not follow the normal distribution. A two-tailed *p* value <0.05 was considered to be statistically significant. All statistical analyses were performed using SPSS 19.0 (SPSS, Inc., Chicago, IL, USA) for windows.

## Results

### Study population

A total of 156 patients were enrolled in the study. Mean age of the entire patients was 59.2 ± 15.5 years. Patients were randomly assigned in a 1:1 ratio to two groups. We excluded 13 patients (6 LL group and 7 standard group patients) due to poor echocardiographic windows. Therefore, data from 143 patients (LL group: n = 72, standard group: n = 71) were finally analyzed.

### Clinical and echocardiographic characteristics

Clinical variables in the LL and standard groups are shown in Table 1. There were no significant differences in age, gender, pacemaker type, pre-implantation QRS duration, comorbidities between the two groups. Furthermore, indications for pacemaker implantation were not statistically significant between the two groups. There were no significant differences in terms of preoperative echocardiographic data between the LL and standard groups.

**Table 1:** Baseline clinical and demographic characteristics of patients

	LL group (n = 72)	Standard group (n = 71)	*p* value
*Demography*			
Age (years)	58.0 ± 15.3	60.3 ± 15.7	0.36
Male (%)	39	44	0.46
Indications (n)			0.68
SSS	48	45	
High degree AVB	24	26	
Pacemaker type (n)			0.71
Single chamber	4	5	
Dual chamber	68	66	
*Comorbidities* (*n*)			
Paroxysmal AF	9	10	0.78
CAD	9	7	0.62
DM	5	6	0.74
Hypertension	18	24	0.25
Pre-QRS width (ms)	96.9 ± 19.2	95.0 ± 17.1	0.57
Pre-echocardiography			
LVEDd (mm)	46.3 ± 5.2	46.5 ± 4.8	0.89
LVESd (mm)	28.8 ± 4.5	28.7 ± 4.3	0.82
LVEF (%)	66.8 ± 5.9	66.0 ± 6.7	0.45
RV diameter (mm)	48.4 ± 5.0	48.9 ± 4.3	0.50
RA diameter (mm)	44.4 ± 4.9	45.8 ± 4.6	0.11

Values are mean ± SD

*SSS* sick sinus syndrome, *AVB* atrial-ventricular block; *AF* atrial fibrillation, *CAD* coronary artery disease, *LVEDd* left ventricular end-diastolic diameter, *LVESd* left ventricular end-systolic diameter, *RV* right ventricular, *RA* right atrial

### Implant procedure and electrical parameters

Lead placement was successful in all cases, with no procedural complications. All tested parameters were within the normal acceptable range for these leads. No significant differences were observed in R-wave amplitude, RV lead impedance, pacing threshold and fluoroscopy time. There was no statistically significant difference in paced QRS duration in patients between LL group compared with standard group (143.8 ± 20.9 vs. 147.2 ± 18.2 ms, *p* = 0.35) (Table 2).

**Table 2:** Pacing data and fluoroscopy time between the LL and standard group

	LL group (n = 72)	Standard group (n = 71)	*p* value
R-wave amplitude (mV)	13.5 ± 5.5	12.1 ± 3.7	0.26
RV threshold (V)	0.64 ± 0.21	0.58 ± 0.19	0.19
RV impedance (Ω)	529 ± 145	536 ± 170	0.80
Paced QRS width (ms)	143.8 ± 20.9	147.2 ± 18.2	0.35
Fluoroscopy time (min)	3.93	3.74	0.14

*LL* left lateral, *RV* right ventricular

### Echocardiographic validation of RVOT pacing sites

TTE of sufficient quality for confirmation of RVOT pacing sites was available in 72 patients in the LL group and 71 patients in the standard group. In the LL group, 60 patients (83 %) were achieved in RVOT septal position. Of the remaining 12 patients, the leads were anchored in the anterior wall in 4 (33 %) and in the free wall in the 8 (67 %). In the standard group, RVOT septal position was observed in 48 patients (68 %). Furthermore, 19 (83 %) patients were classified as being positioned on the anterior wall and 4 (17 %) as being on the free wall. There were significant differences in RVOT septal pacing between the LL and standard groups (*p* = 0.029). The position of RVOT anterior wall in LL group was significantly less than standard group (*p* = 0.001). No significant difference in RVOT free wall pacing was shown between 2 groups (*p* = 0.239) (Table 3).

**Table 3:** Comparison of RVOT pacing sites between the LL and standard group

RVOT pacing site	LL group (n = 72)	Standard group (n = 71)	*p* value
Septal	60 (83.3)	48 (67.6)	0.029
Anterior wall	4 (5.6)	19 (26.8)	0.001
Free wall	8 (11.1)	4 (5.6)	0.239

*LL* left lateral; *RVOT* right ventricular outflow tract

### ECG characteristics

The ECG characteristics are shown in Table 4. RVOT septal pacing was associated with a shorter QRS duration compared with RVOT non-septal pacing (*p* = 0.015). QRS vector in lead I was found more frequently negative voltage in septal pacing than in non-septal pacing (*p* < 0.001). There was no significant difference in the presence of notching of QRS complex in inferior leads or QRS transition zone.

**Table 4:** ECG characteristics of patients

	Septum (n = 108)	Non-septum (n = 35)	*p* value
QRS duration (ms)	142.8 ± 19.1	152.6 ± 19.4	0.015
q in lead I (%)	81 (75.0)	12 (34.3)	<0.001
Notching in inferior leads	8 (7.4)	6 (17.1)	0.092
Transition zone	4.5 ± 1.0	5.1 ± 0.88	0.153

The transition zone was defined as the first precordial lead where the R wave was higher than the S wave

## Discussion

Our randomized prospective study demonstrates that only 68 % of patients were achieved in RVOT septal position in the standard group, but 83 % of patients were found achieved in RVOT septal position in the LL group. Therefore, the LL fluoroscopic view could provide useful information that help confirm RVOT septal pacing site and the standard fluoroscopic technique may not be adequate for the correct documentation of pacing lead position for routine clinical practice especially when attempting RVOT septal pacing.

Long-term RV apical pacing is associated with adverse effects on left ventricular function [18, 19]. RVOT septal pacing has been advocated as a substitute for RV apical pacing due to a more physiological left ventricular (LV) activation and less dyssynchrony [20]. The fluoroscopic criterion has been established on correct assessment of RVOT septal lead placement [6]. On the basis of the radiographic criteria, the RVOT septal position is considered to be reachable in the majority of studies [13, 21–23]. The septum lies posteriorly with the free wall in front, and separating them is the anterior wall. The conventional fluoroscopic views are difficult to differentiate between RVOT septal and anterior wall positions. However, the LL fluoroscopic view could clearly define the anterior-posterior plane (Fig. 1).Our study showed that the position of RVOT anterior wall in LL group was significantly less than standard group. Therefore, using the fluoroscopic criterion for placing on the RVOT septum, the lead might position on the anterior wall instead of septum [24]. However, pacing from anterior wall should be avoided as it may result in adverse effects such as reduced LV ejection fraction [12], cardiac tamponade [25], or might carry a risk for damage of the left anterior descending artery [26]. LL view may allow less localization of the lead in the RVOT anterior wall.

The placement success rate in RVOT septum based on the conventional fluoroscopic criterion has been addressed in several studies. In a report of RVOT pacing, only 61 % of the leads was shown to be on the septum using standard fluoroscopic projection [15]. Domenichini et al. [13] randomised 59 patients in apical or septal pacing. The exact location of the RV lead was determined using TTE. The septal position was only observed in 54 % of patients, the anterior position was found in the remaining 46 % of patients. Ng et al. [12] studied 55 patients in apical or septal pacing. They also found that despite the standard fluoroscopic views for placing the lead on septum, the final position was heterogeneous. The septal pacing site was achieved in 70.6 % of patients. Osmancik et al. [27] reported that the RV lead of 51 patients was implanted into RVOT septum according to the standard criteria. The exact position of the lead tip was access using cardiac computed tomography. The RV lead was anchored in the RVOT septum in 41 % of patients and in the anterior wall in the remaining of 59 %.

These above-mentioned findings are in consistent with our result. In the present study, the conventional fluoroscopic guidance had a low accuracy in identifying RVOT septal pacing. However, pacing at the RVOT septal pacing could achieved in 83 % of patients in guidance with standard fluoroscopic and LL radiographic views. The success rate of septal placement increased from 68 % to 83 %. McGavigan et al. [15] studied 56 patients which had LL radiographys performed. The authors found that a posterior projection of the lead tip on the LL fluoroscopic view had a high specificity for septal lead placement. Pang et al. [28] retrospectively analyzed 60 patients whose lead position was determined by computed tomography. Their result showed that the lead of 6 cases pointed to the spine in the LL projection. Of these, the lead tip of 5 patients located on the septum, and only one was on the anterior RV wall. Therefore, it is reasonable that LL fluoroscopic view may be employed in order to confirm septal position more correctly in the future.

In our study, the RVOT septal pacing produced a significantly narrower QRS duration than non-septal pacing. This reduction in QRS duration suggests a shorter total ventricular activation time and greater ventricular synchrony, which might help decrease adverse remolding [4, 23]. Therefore, true RVOT septum is a desirable pacing site at the level of electrophysiology. The presence of q waves or negative QRS in lead I, which is the most common characteristic ascribed to septal pacing, was also more frequent in pacing from true septal pacing.

### Study limitations

We did not use cardiac computed tomography to confirm the lead position. It was not available in sufficient numbers of patients enrolled in the present study. We did not perform a clinical follow-up. A clinical follow-up, including echocardiography to evaluate LV function and dyssynchrony of groups with leads in different RVOT positions should be the next step.

## Conclusions

We conclude that the standard fluoroscopic technique may not be adequate for the correct documentation of RVOT septal pacing lead position. LL fluoroscopic view may provide important information for correct documentation of RVOT septal placement.
